# Increased tau-induced inflammatory responses are associated with a greater degree of atherosclerosis in progressive supranuclear palsy

**DOI:** 10.3389/fnagi.2025.1608631

**Published:** 2025-08-11

**Authors:** Yi-Xin He, Rui Zhang, Chen Xie, Si-Fan Ji, Wen-Jing Deng, Jun-Fang Teng

**Affiliations:** ^1^Department of Neurology, The First Affiliated Hospital of Zhengzhou University, Zhengzhou, Henan, China; ^2^Henan Neurological Disease Medical Center, The First Affiliated Hospital of Zhengzhou University, Zhengzhou, Henan, China; ^3^Office of Citizen Affair, The Third Affiliated Hospital of Zhengzhou University, Zhengzhou, Henan, China

**Keywords:** progressive supranuclear palsy, atherosclerosis, inflammation, tau, macrophage

## Abstract

**Introduction:**

It has been reported that progressive supranuclear palsy (PSP) has a higher prevalence of cerebrovascular disease (CVD); however, the relationship between PSP and atherosclerotic disease remains poorly understood. This study aimed to evaluate the burden of atherosclerosis in patients with PSP and explore the potential role of tau-induced inflammation in its pathogenesis.

**Methods:**

We conducted a cross-sectional study involving 56 patients with PSP and 56 age- and sex-matched healthy controls. Carotid and cerebral atherosclerosis was assessed using ultrasound and magnetic resonance angiography, including measurements of maximum carotid intima-media thickness (max-CIMT), maximum carotid plaque thickness (max-CPT), total plaque number (TPN), carotid plaque score (CPS), maximum carotid stenosis percentage, and global stenosis score (GSS). Plasma levels of traditional atherosclerosis risk factors, total tau, phosphorylated tau (p-tau181, p-tau396), inflammatory cytokines and macrophage proportions were measured. To further investigate the mechanism, we established a mouse model using repeated tail vein injections of tau-preformed fibrils (tau-PFFs), followed by histological and biochemical analysis after 3 months.

**Results:**

Compared to controls, patients with PSP exhibited significantly higher atherosclerotic burden across most measured vascular parameters. Plasma levels of total tau, p-tau181, macrophage proportions and pro-atherogenic inflammatory markers were elevated in patients with PSP. Among them, elevated interleukin-6 (IL-6) levels were positively correlated with the atherosclerosis severity and total tau levels in patients with PSP. In the tau-PFFs mouse model, localized thickening of the aortic wall, increased circulating macrophages, and enhanced inflammatory responses in both plasma and vascular tissues were observed at 3 months post-injection.

**Conclusion:**

Our findings suggest that patients with PSP reveals increased atherosclerotic burden, potentially mediated by tau-induced systemic inflammation and macrophage activation. Monitoring tau levels and inflammatory markers may serve as a valuable approach for assessing vascular risk in patients with PSP. Furthermore, targeting tau-related inflammatory pathways may offer a novel therapeutic strategy for mitigating atherosclerosis in this population.

## 1 Introduction

Progressive supranuclear palsy is the most common atypical Parkinsonian disorder, characterized by supranuclear gaze palsy, axial rigidity, postural instability, and cognitive dysfunction as its main clinical features (Steele et al., 1964). With a median survival of approximately 7–8 years, PSP causes a rapid decline in the quality of life (Chiu et al., 2010). Currently, there are no effective treatments for PSP. The prevalence of PSP is estimated to be 5–6 per 100,000 people worldwide, with symptoms usually appearing after age 60 (Parthimos and Schulpis, 2020). PSP primarily affects older individuals, who often suffer from a variety of other chronic diseases, such as cerebrovascular disease.

Cerebrovascular disease is the leading cause of long-term disability in adults and the third leading cause of death worldwide (Wang et al., 2020; GBD 2021 Causes of Death Collaborators, 2024). Ischemic stroke accounts for 85% of all CVD cases, with atherosclerotic lesions being the most common cause (Wu et al., 2019). Carotid color Doppler ultrasound (US) and magnetic resonance angiography (MRA) are widely used to assess the carotid and intracranial arteries for atherosclerosis, providing non-invasive vascular imaging without radiation or the increased risk of conventional angiography (Iino et al., 2019; Chen et al., 2024).

Studies on the association between PSP and atherosclerotic diseases are limited. In a previous study, CVD was found to be a characteristic prediagnostic comorbidity of PSP (Kwasny et al., 2021). A cross-sectional study of comorbidities and co-medications in patients with PSP and no neurodegenerative diseases also found that the overall prevalence of cardiovascular and cerebrovascular diseases was higher in patients with PSP (Greten et al., 2023). In the present study, we aimed to investigate the differences in atherosclerotic burden between the PSP and control groups using carotid-color Doppler ultrasound and MRA, as well as explore the possible reasons for the development of the differences.

Progressive supranuclear palsy is a primary tauopathy characterized by widespread accumulation of hyperphosphorylated tau, particularly the 4-repeat (4R) isoform, in neurons and glial cells (DeRosier et al., 2024). Genetic studies have linked PSP with polymorphisms and mutations in the MAPT gene, which encodes the microtubule-associated protein tau (Ressler et al., 2024). Tau aggregates have been shown to activate microglia, thereby promoting the release of pro-inflammatory cytokines such as IL-6, IL-1β and TNF-α (Gao et al., 2023; Rice et al., 2024). In addition to central immune activation, tau may stimulate the proliferation of macrophages and enhance inflammatory responses in peripheral tissues (Chen et al., 2023). It’s worth noting that an experimental study showed that inhibition of tau protein phosphorylation by means of an intervention ameliorated atherosclerotic lesions (Yang et al., 2023). Based on this background, we hypothesize that tau protein may be associated with atherosclerosis. We measured plasma tau protein levels and the levels of circulating atherosclerosis-related inflammatory mediators in patients with PSP and in the control group, in order to explore potential links between tau and vascular inflammatory pathways.

The intracerebral injection of tau-preformed fibrils (tau-PFFs) is a common method of modeling tau aggregation and multiplication *in vivo* in tauopathies (Iba et al., 2013). Notably, tau seeds injected via peripheral routes–including intravenous and intraperitoneal–can induce central pathology through prion-like mechanisms (Clavaguera et al., 2014; Houben et al., 2020). These findings raise the possibility that repeated peripheral exposure to tau-PFFs may similarly elicit inflammatory and vascular responses relevant to tauopathies such as PSP. Here, we established a novel peripheral tauopathy model by administering 5 μg of tau-PFFs via tail vein every 2 weeks, aiming to mimic chronic peripheral tau exposure and investigate its effect on systemic inflammation and vascular pathology.

## 2 Materials and methods

### 2.1 Participants

Patients with PSP (*n* = 56) aged > 50 years were recruited from the Department of Neurology at the First Affiliated Hospital of Zhengzhou University. Diagnoses of probable or possible PSP were made according to the MDS criteria by experienced specialists in neurodegenerative diseases who were unaware of laboratory results (Höglinger et al., 2017). Age- and sex-matched healthy controls (HCs) (*n* = 56) with no history of neurodegenerative disease were recruited from the Physical Examination Center of the First Affiliated Hospital of Zhengzhou University. The exclusion criteria for both groups were as follows: (1) use of antibiotics or immunosuppressants within 3 months prior to sample collection; and (2) complications such as recent or persistent inflammatory diseases, autoimmune diseases, or malignant tumors. The participants underwent a detailed clinical interview and a complete neurological examination. Demographic and clinical information, including age, sex, stroke risk factors, disease duration, and years of education were collected at the time of sampling. Participants underwent a physical examination and a battery of laboratory tests, including routine blood tests for coagulation, C-reactive protein (CRP), blood glucose and lipids, liver and kidney function, and homocysteine, as well as carotid ultrasound and MRA. Disease severity was assessed using the modified Hoehn and Yahr (H&Y) staging scale (Goetz et al., 2004), a Movement Disorder Society-sponsored revision of the Unified Parkinson’s Disease Rating Scale, Part III (UPDRS-III) (Goetz et al., 2008), and the Progressive Supranuclear Palsy Rating Scale (PSPRS) (Golbe and Ohman-Strickland, 2007). The Mini-Mental State Examination (MMSE) (Folstein et al., 1975) was used to assess cognitive performance. This study was approved by the Ethics Committee of the First Affiliated Hospital of Zhengzhou University (2024-KY-0046-002), and informed consent was obtained from each participant.

### 2.2 Human sample collection

Peripheral blood samples were collected in endotoxin-free K2 EDTA 10-mL tubes after overnight fasting. The samples were immediately placed on ice and centrifuged at 2,000 rpm for 10 min at 4°C to obtain plasma samples. These samples were then stored at −80°C until biochemical analysis if immediate detection was not possible.

### 2.3 Carotid artery assessment

To assess carotid atherosclerosis, we examined the bilateral carotid arteries of all participants using color Doppler ultrasound with a 7.5 MHz linear probe (GE Healthcare, Horten, Norway). Max-CIMT is the mean of the maximum left and right carotid intima-media thicknesses, which is defined as the distance from the luminal intimal interface to the medial-adventitial interface in a plaque-free area located 1–1.5 cm below the level of the distal branches of the common carotid artery. Carotid plaque was defined as a focal area of wall thickening or protrusion within the arterial lumen of any carotid artery segment (common carotid artery, bifurcation, and internal and external carotid arteries) greater than 50% of the surrounding wall thickness. Maximum carotid plaque thickness (max-CPT) (Walker et al., 2009) was measured at the most prominent point of the plaque in the multi-angle image of the carotid artery or at the maximum wall thickness in the absence of carotid plaques. The total carotid plaque number (TPN) was calculated as the sum of all plaque numbers in the common carotid artery as well as the internal and external carotid arteries. The carotid plaque score (CPS) was calculated using ultrasound according to the methodology used in the prospective ACE 1950 (Akershus Cardiac Examination 1950 study) (Ihle-Hansen et al., 2018). Each side of the carotid artery was divided into the following four segments: common carotid artery, carotid bifurcation, internal carotid artery, and external carotid artery. Plaque measurements were taken in cross-sections for each segment, and the thickness of the largest plaque in each segment was recorded and scored according to maximum diameter (plaque diameters of ≥1.5, ≥2.5, and ≥3.5 mm were scored 1, 2, and 3, respectively), with occluded segments scored as 3. Finally, the scores for all the segments on both sides were summed to obtain an overall CPS ranging from 0 to 24. The percentage of maximal stenosis (stenosis%) was calculated by measuring the residual luminal internal diameter at the site of maximal stenosis and the original internal diameter, then dividing the difference by the original internal diameter (Liu et al., 2022). The operator was blinded to the medical histories of the participants.

### 2.4 Magnetic resonance angiography examination

All MRA scans were performed using a 3 Tesla magnetic resonance imaging (MRI) scanner (Siemens Healthcare, Erlangen, Germany). MRI of intracranial arteries using three-dimensional time-of-flight gradient echo to determine the vascular territory and extent of intracranial stenosis (ICS). We recorded the number of participants with stenosis and the degree of stenosis at the narrowest point in each of the 11 predefined vascular territories, including the left and right internal carotid arteries (ICA), left and right middle cerebral arteries (MCA), left and right anterior cerebral arteries (ACA), left and right posterior cerebral arteries (PCA), left and right vertebral arteries (VA), and basilar arteries (BA). Using the criteria established in the Warfarin-Aspirin Symptomatic Intracranial Disease Trial, the degree of stenosis of the intracranial arteries was graded into five levels: 0 (normal; no detectable stenosis), 1 (mild; <50% stenosis), 2 (moderate; 51%–70% stenosis), 3 (severe; 71%–99% stenosis), and 4 (occlusion) (Samuels et al., 2000). The total score for the intracranial arteries was calculated as the global stenosis score (GSS), and the degree of stenosis was determined by the consensus of at least two neurologists.

### 2.5 Animals

C57BL/6J male mice were obtained from the Experimental Animal Center of Zhengzhou University. The mice were housed on a 12-h light/dark cycle with *ad libitum* access to food and water. Each mouse received 3–7 g/day of food and 4–7 mL/day of purified water. All procedures were performed in accordance with the Guide for the Care and Use of Laboratory Animals. The Ethics Committee of the First Affiliated Hospital of Zhengzhou University approved the study protocol and procedures (2024-KY-0046-002).

### 2.6 Tau-preformed fibrils (tau-pFFs) preparation

Recombinant mouse tau monomers with four structural domains (CSB-YP013481MO, CUSABIO, Wuhan, China) were co-incubated with heparin at a concentration of 1 mg/mL in a buffer (10 mM HEPES, pH 7.4, 100 mM NaCl, 5 mM dithiothreitol) (Frost et al., 2009). The mixture was shaken at 1,000 rpm for 10 days at 37°C to obtain tau-PFFs. After fragmentation using an ultrasonic crusher, the PFFs were stored at −80°C for backup.

### 2.7 Tail vein injection and sampling

At 6–8 weeks of age, mice (12 per group) received tail vein injections every 2 weeks with either 5 μg of tau-PFFs (25 μL, 0.2 mg/mL) or an equal volume (25 μL) of sterile phosphate-buffered saline (PBS) as a control. Tissues were harvested 3 months after the first injection. Following anesthesia via intraperitoneal injection of pentobarbital (10 mg/mL, 50 mg/kg), mice were euthanized for sample collection. Blood (0.5 mL) was obtained from the right ventricle of mice. Fresh blood (0.5 mL per mouse) from 6 mice per group immediately subjected to flow cytometry analysis, and the blood samples (0.5 mL per mouse) from the remaining 6 mice centrifuged at 3,500 rpm for 15 min to collect plasma for storage at −80°C and subsequent ELISA. All mice then underwent PBS perfusion. Aortic arches were carefully dissected, with 6 fresh specimens per group flash-frozen in dry ice for Western blot analysis and the others fixed in 4% paraformaldehyde for frozen sectioning.

### 2.8 Histopathology analysis

Aortic tissues were fixed in 4% paraformaldehyde (PFA) for 24 h at 4°C, then cryoprotected by immersion in 30% sucrose solution (w/v in PBS) for an additional 24 h at 4°C until the tissues sank to the bottom of the vial. Samples were embedded in optimal cutting temperature (OCT) compound (Tissue-Tek, Sakura Finetek, USA), rapidly frozen on dry ice, and stored at −80°C until sectioning. For histopathological analysis, 20 μm-thick frozen sections were cut using a cryostat (Leica CM1950, Germany) at −20°C, mounted onto poly-L-lysine-coated glass slides, and air-dried for 1 h at room temperature. Sections were subsequently stained with hematoxylin and eosin (H&E) or Oil Red O (Solarbio, Beijing, China) according to the manufacturer’s protocols. For immunohistochemistry, frozen tissue sections were peroxidase blocked, followed by blocking with normal goat serum for 1 h. Subsequently, they were incubated with CD68 primary antibody (ab955, Abcam, 1:500, mouse) overnight at 4°C. After rinsing with PBS, the sections were incubated with horseradish peroxidase-conjugated secondary antibodies (ab6789, Abcam, 1:500) for 2 h at room temperature. The sections were then washed thrice for 5 min each and stained with 3,3′-diaminobenzidine. The images were captured using a DP74 camera (Olympus, Tokyo, Japan).

### 2.9 Western blot

Fresh aortic tissue was isolated, snap-frozen with solid carbon dioxide, and homogenized in RIPA lysis buffer containing a protease inhibitor mixture (Beyotime, Shanghai, China). Homogenized fractions were centrifuged at 120,000 *g* for 30 min at 4°C, and the supernatant was collected. Fractions of 10 μL were separated by electrophoresis on 8% or 12% polyacrylamide gels and blotted onto polyvinylidene fluoride membranes (Millipore, Darmstadt, Germany). The membranes were blocked with 5% non-fat dry milk in TBST (Tris-buffered saline, containing 0.1% Tween-20) for 1 h at room temperature and then incubated overnight at 4°C in TBST diluted with primary antibodies. After several rinses with TBST, the membranes were incubated with horseradish peroxidase-conjugated secondary antibodies for 1 h at room temperature. Finally, the membranes were detected using enhanced chemiluminescence (Thermo Fisher Scientific). Protein expression levels were normalized to GAPDH expression levels and analyzed using the ImageJ software (v1.53, National Institutes of Health, USA).

The primary and secondary antibodies used were as follow: CD68 (ab955, Abcam, 1:500, mouse), Tau (MN1000, Invitrogen,1:500, mouse), interleukin-1beta (IL-1β, ab200478, Abcam, 1:500, rabbit), tumor necrosis factor-alpha (TNF-α, 17590-1-AP, Proteintech, 1:500, rabbit), interleukin-6(IL-6, DF6087, Affinity, 1:500, rabbit), glyceraldehyde-3-phosphate dehydrogenase (GAPDH, 10494-1-AP, Proteintech, 1:2000, rabbit), Anti-Mouse IgG H&L (ab6789, Abcam, 1:2000), Anti-Rabbit IgG H&L (ab6721, Abcam, 1:2000).

### 2.10 Enzyme-linked immunosorbent assay (ELISA)

Following specimen collection, plasma biomarker levels were quantified using ELISA according to the manufacturer’s protocols. The following ELISA kits were used: Human interleukin-6 (IL-6) ELISA kit (E-HSEL-H0003, Elabscience Biotechnology, Wuhan, China), Human interleukin-1β (IL-1β) ELISA kit (E-HSEL-H0001, Elabscience Biotechnology, Wuhan, China), Human interleukin-10 (IL-10) ELISA kit (E-EL-H0005, Elabscience Biotechnology, Wuhan, China), Human interferon γ (IFN-γ) ELISA kit (E-HSEL-H0007, Elabscience Biotechnology, Wuhan, China), Human tumor necrosis factor-α (TNF-α) ELISA kit (E-HSEL-H0109c, Elabscience Biotechnology, Wuhan, China), Human total tau ELISA kit (ml057755, mlbio, Shanghai, China),and Human P-tau181 ELISA kit (ml057691, mlbio, Shanghai, China), Human P-tau396 ELISA kit (E-EL-H5314c, Elabscience Biotechnology, Wuhan, China), Mouse interleukin-6 (IL-6) ELISA kit (ml098430, mlbio, Shanghai, China), Mouse interleukin-1β (IL-1β) ELISA kit (ml098416, mlbio, Shanghai, China) and Mouse tumor necrosis factor-α (TNF-α) ELISA kit (mIC50536-1, mlbio, Shanghai, China). All procedures were performed according to the manufacturer’s instructions.

### 2.11 Flow cytometry

For humans, 3 mL of anticoagulated whole blood was collected from each participant. Red blood cells (RBCs) were osmotically lysed with erythrocyte lysis buffer (Solarbio, Beijing, China), washed with PBS after lysis, and 1–10 × 10^6^ cells were resuspended in a diluted 100 μL Zombie Aqua™ solution (1:100, BioLegend, USA). After 10 min incubation, the cells were resuspended, washed with PBS, and incubated with anti-human CD45 (1:100, BioLegend, USA, 2D1, APC/Cyanine7), anti-human CD14 (1:100, BioLegend, USA, HCD14, FITC), anti-human CD86 (1:100, BioLegend, USA, IT2.2, PE), and anti-human CD206 (1:100, BioLegend, USA, 15-2, Pacific BLue™) for 30 min at 4°C in the dark. After washing, fixation breakers (eBioscience, USA) were added, and the cells were incubated for 30 min at 4°C in the dark. After washing, the cells were incubated with anti-human CD68 (1:100, BioLegend, USA, Y1/82A, Alexa FLuor^®^ 647) for 30 min at 4°C in the dark. After washing, flow cytometry was performed using BD FACS Celesta.

For mice, blood was collected from the right ventricle and placed in a 1.5-mL anticoagulant tube. It was then incubated with anti-mouse CD45 (1:100, BD Pharmingen, USA, 30-F11, PE), anti-mouse CD11b (1:100, BD Pharmingen, USA, M1/70, APC), and anti-mouse F4/80 (1:100, Invitrogen, USA, BM8, Alexa FLuor™ 488) for 30 min in the dark at 4°C. RBCs were lysed by adding erythrocyte lysis buffer (Solarbio, Beijing, China) for 10 min at room temperature and then centrifuged at 350 *g* for 5 min. After washing, flow cytometry was performed using BD FACS Celesta.

Flow cytometry data were analyzed using FlowJo software (v10.4, FlowJo LLC, USA).

### 2.12 Statistical analyses

Statistical analyses were performed using the IBM SPSS Statistics (version 27.0, IBM Corp., Armonk, NY, USA) and GraphPad Prism 9.0 software (GraphPad Software Inc., San Diego, CA, USA). The chi-square test was used to compare proportions. Continuous variables were described as means ± standard deviation or median and interquartile range, depending on the result of a D’Agostino-Pearson normality test, and compared using the unpaired Student’s *t*-test or Mann–Whitney U test, respectively. Analysis of covariance (ANCOVA) was applied to compare group differences while adjusting for potential confounders (e.g., age, gender, smoking status and stroking history). Multivariable linear regression analysis was performed to examine the associations between independent variables (*X*-axis) and dependent variables (*Y*-axis), adjusting for potential confounders including age, sex, smoking status, hypertension, diabetes, dyslipidemia and stroking history. A *p*-value of < 0.05 was considered statistically significant.

## 3 Results

### 3.1 Patients with PSP revealed a greater atherosclerotic burden than HCs

The demographic and clinical information of the patients in the two groups are summarized in Table 1. A total of 56 HCs and 56 patients with PSP were enrolled in this study. There were no significant differences observed in atherosclerosis risk factors, including age, gender, hypertension, diabetes, dyslipidemia, and smoking, between HCs and patients with PSP. However, the prevalence of stroke was significantly higher in patients with PSP than in HCs (39.29% vs. 16.07%, *p* = 0.011), which is similar to a previous report (Greten et al., 2023).

**TABLE 1:** Basic clinical characteristics of subjects.

Clinical characteristics	HCs (*n* = 56)	PSP (*n* = 56)	*P*-value
Age, years	65.05 ± 7.96	65.16 ± 8.47	0.921
Female, *n* (%)	17 (30.36)	17 (30.36)	>0.999
Smoking, *n* (%)	14 (25)	12 (21.43)	0.823
Hypertension, *n* (%)	23 (41.07)	21 (37.50)	0.847
Diabetes, *n* (%)	7 (12.50)	11 (19.64)	0.441
Dyslipidemia, *n* (%)	6 (10.71)	6 (10.71)	>0.999
Stroke, *n* (%)	9 (16.07)	22 (39.29)	0.011*
Estimated disease duration, mo	–	33 (18.5, 48)	–
H&Y stage	–	2.63 ± 1.05	–
UPDRS-III	–	27.32 ± 7.26	–
PSPRS	–	31 (27, 35)	–
MMSE	–	26 (24, 27)	–
Education, years	–	7.13 ± 3.87	–

Data are presented as mean ± standard deviation (SD), median (P25, P75) or *n* (%), (*n* = 56 vs. *n* = 56), compared by two-tailed unpaired *t*-test or Mann–Whitney U test, or chi-square test. HCs, health controls; PSP, Progressive supranuclear palsy; H&Y stage, Hoehn and Yahr stage scale; UPDRS-III: Unified Parkinson’ Disease Rating Scale, part III; PSPRS: Progressive Supranuclear Palsy Rating Scale; MMSE: Mini-Mental State Examination.

*P < 0.05, presents a significant difference.

**FIGURE 1:**
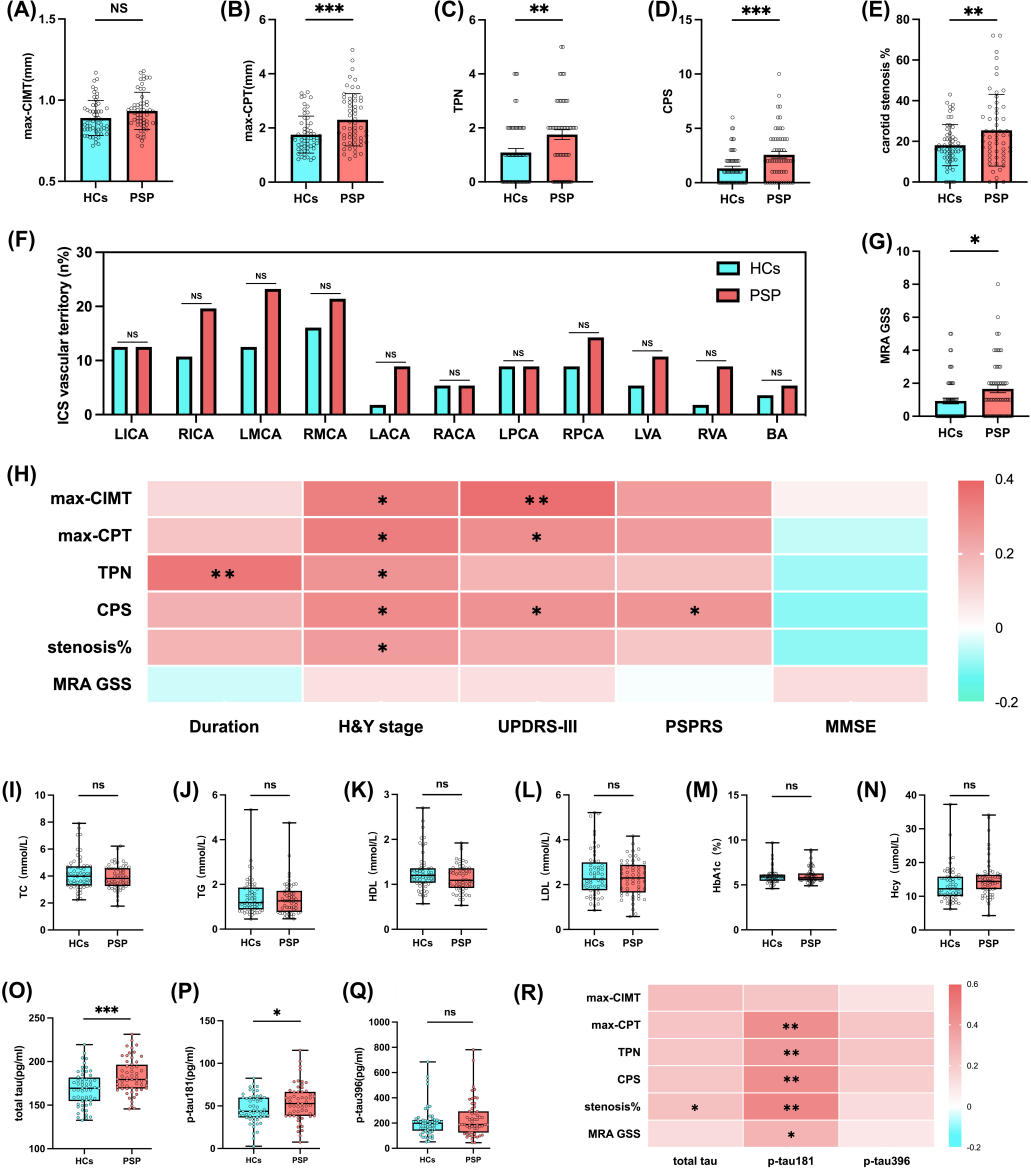
Greater atherosclerotic burden and elevated plasma total tau, p-tau181 levels in patients with PSP. Using carotid artery Doppler ultrasound to assess the degree of carotid artery atherosclerosis by evaluating **(A)** max-CIMT, **(B)** max-CPT, **(C)** TPN, **(D)** CPS, and **(E)** carotid stenosis% in HCs (*n* = 56) and patients with PSP (*n* = 56). Using MRA to evaluate the degree of intracranial arterial atherosclerosis by recording **(F)** ICS vascular territory and **(G)** MRA GSS in HCs (*n* = 56) and patients with PSP (*n* = 56). **(H)** Heatmap of multivariable linear regression heatmap showing the association between disease duration/clinical scores H&Y staging scale, UPDRS-III, PSPRS, MMSE and atherosclerosis measures (max-CIMT, max-CPT, TPN, CPS, carotid stenosis% and MRA GSS) in patients with PSP (*n* = 56). Peripheral **(I–L)** blood lipid, **(M)** glycosylated hemoglobin, **(N)** homocysteine parameters, and plasma levels of **(O)** total tau, **(P)** p-tau181, **(Q)** p-tau396 in HCs (*n* = 56) and patients with PSP (*n* = 56). **(R)** Heatmap of multivariable linear regression heatmap showing the association between tau levels (total tau, p-tau181, p-tau396) and atherosclerosis measures (max-CIMT, max-CPT, TPN, CPS, carotid stenosis% and MRA GSS) in patients with PSP (*n* = 56). Results are expressed as mean and SD. Statistical approaches: **(A–E, G, I–Q)** covariance (ANCOVA), with age, gender, smoking status and stroke history as covariates, **(F)** chi-square test, **(H, R)** multivariable linear regression. In heatmaps, each cell represents the standardized regression coefficient (β) derived from multivariable linear regression models, where each *Y*-axis was regressed on *X*-axis while adjusting for age, gender, smoking history, hypertension, diabetes, and dyslipidemia (For MMSE, one more correction for period of education). The color intensity of heatmap indicates the strength and direction of association (β), with positive associations shown in red and negative in blue. *P* < 0.05 indicates statistical significance (*p < 0.05, **p < 0.01, ***p < 0.001), and ns indicates *p* ≥ 0.05.

In this study, we evaluated systemic atherosclerotic burden comprehensively by assessing extracranial and intracranial vascular lesions using a multimodal approach. For extracranial assessment, carotid ultrasound provided several key indices: maximum carotid intima-media thickness (max-CIMT), which measures arterial wall thickening as an early marker of generalized atherosclerosis; maximum carotid plaque thickness (max-CPT), reflecting the most severe focal plaque deposition; total plaque number (TPN) indicating the extent of multifocal plaque involvement; and carotid plaque score (CPS), a composite metric integrating plaque size and number to represent overall plaque burden. We additionally quantified the percentage of carotid stenosis to determine the degree of stenosis. For intracranial assessment, we calculated the proportion of intracranial stenosis (ICS) vascular territory to determine the spatial extent of flow-limiting lesions within specific brain-supplying arteries. Finally, the magnetic resonance angiography (MRA)-based global stenosis score (GSS) was employed as a comprehensive metric that simultaneously evaluates the severity and distribution of stenotic lesions across intracranial arterial trees. This multidimensional approach enabled precise characterization of atherosclerosis at different vascular levels and pathophysiological stages, from early wall thickening to advanced flow-limiting stenoses. Patients with PSP exhibited significantly elevated values across multiple vascular parameters compared to HCs, suggesting a greater burden of both systemic and cerebral atherosclerosis. Specifically, after adjusting for age, gender, smoking status and stroke history, the PSP group exhibited significantly higher max-CPT (2.01 mm vs. 1.59 mm, *p* < 0.001), TPN (median 2 vs. 1, *p* = 0.005), CPS (median 2 vs. 1, *p* < 0.001), and carotid stenosis percentage (22.5% vs. 16.0%, *p* = 0.004) compared to HCs; however, max-CIMT (0.93 mm vs. 0.89 mm, *p* = 0.065) did not differ significantly between the groups (Figures 1A–E and Table 2). Another aspect, although intracranial stenosis involvement in individual vascular territories was more frequent in the PSP group, though not statistically significant (Figure 1F and Table 2); in contrast, MRA GSS reflecting overall stenosis severity, was significantly elevated in PSP patients (median 1 vs. 0, *p* = 0.041) after covariate adjustment (Figure 1G and Table 2). This finding is reasonable, given that the GSS reflects the severity of overall stenosis rather than the number of lesions.

**TABLE 2:** Comparison of ultrasound and MRA-based atherosclerotic vascular measures between HCs and patients with PSP.

Variable	HCs (*n* = 56)	PSP (*n* = 56)	*P*-value
Max-CIMT, mm	0.89 ± 0.11	0.93 ± 0.11	0.065
Max-CPT, mm	1.59 (1.26, 2.15)	2.01 (1.52, 3.01)	<0.001*
TPN	1 (0, 2)	2 (1, 2.75)	0.005*
CPS	1 (0, 2)	2 (1, 4)	<0.001*
Carotid stenosis%	16 (12, 22.75)	22.5 (13.25, 35.5)	0.004*
**ICS vascular territory, n (%)**			
Left ICA	7 (12.50)	7 (12.50)	>0.999
Right ICA	6 (10.71)	11 (19.64)	0.292
Left MCA	7 (12.50)	13 (23.21)	0.217
Right MCA	9 (16.07)	12 (21.42)	0.629
Left ACA	1 (1.79)	5 (8.93)	0.206
Right ACA	3 (5.36)	3 (5.36)	>0.999
Left PCA	5 (8.93)	5 (8.93)	>0.999
Right PCA	6 (10.71)	8 (14.29)	0.557
Left VA	3 (5.36)	6 (10.71)	0.489
Right VA	1 (1.79)	5 (8.93)	0.206
BA	2 (3.57)	3 (5.36)	>0.999
MRA GSS	0 (0, 1)	1 (0, 2)	0.041*

Data are presented as mean ± standard deviation (SD), median (P25, P75) or *n* (%), (*n* = 56 vs. *n* = 56), compared by chi-square test or covariance (ANCOVA), with age, gender, smoking status and stroke history as covariates. HCs, health controls; PSP, Progressive supranuclear palsy; max-CIMT, maximum carotid intima-media thickness; max-CPT, maximum carotid plaque thickness; TPN, total carotid plaque number; CPS, carotid plaque score; stenosis%, percentage of maximum carotid stenosis; MRA GSS, magnetic resonance angiography global stenosis score; ICS, intracranial stenosis; ICA, internal carotid arteries; MCA, middle cerebral arteries; ACA, anterior cerebral arteries; PCA, posterior cerebral arteries; VA, vertebral arteries; BA, basilar arteries.

*P < 0.05, presents a significant difference.

To investigate the association between the severity of atherosclerosis and disease progression in patients with PSP, we performed regression analyses with atherosclerotic measures as dependent variables and disease duration/clinical severity scales as independent variables. Potential confounding factors, including age, gender, smoking status, hypertension, diabetes, dyslipidemia, and stroke history, were adjusted for in the analyses (for MMSE, one more correction for period of education). In adjusted multivariate models, longer disease duration was independently associated with higher TPN (β = 0.348, *p* = 0.004). H&Y stage exhibited positive associations with max-CIMT (β = 0.328, *p* = 0.015), max-CPT (β = 0.331, *p* = 0.012), TPN (β = 0.272, *p* = 0.043), CPS (β = 0.300, *p* = 0.016), and carotid stenosis% (β = 0.256, *p* = 0.036), whereas UPDRS-III correlated with max-CIMT (β = 0.371, *p* = 0.007), max-CPT (β = 0.288, *p* = 0.033), and CPS (β = 0.278, *p* = 0.030), and PSPRS solely with CPS (β = 0.270, *p* = 0.036) (Figure 1H and Supplementary Table 1).

Overall, these findings indicate that individuals with PSP may exhibit a more pronounced atherosclerotic vascular profile, with a positive correlation observed between certain atherosclerotic indicators and disease severity. These vascular changes may provide a pathophysiological link between systemic atherosclerosis and neurodegeneration in PSP, supporting the potential role of vascular biomarkers in disease assessment.

### 3.2 Higher plasma total tau and p-tau181 levels in patients with PSP correlate with atherosclerosis severity

To explore the reasons for the increased susceptibility of patients with PSP to atherosclerosis, we examined traditional atherosclerosis risk factors such as total cholesterol (TC), triglycerides (TG), low-density lipoprotein cholesterol (LDL), high-density lipoprotein cholesterol (HDL), hemoglobin A1c (HbA1c), and homocysteine (Hcy) levels in the plasma. However, we found no significant differences in the levels of TC, TG, LDL, HDL, HbA1c, or Hcy between the two groups (Figures 1I–N and Table 3).

**TABLE 3:** Comparison of plasma metabolic, tau, and inflammatory markers between HCs and patients with PSP.

Variable	HCs (*n* = 56)	PSP (*n* = 56)	*P*-value
TC, mmol/L	3.99 (3.30, 4.72)	3.93 (3.27, 4.59)	0.280
TG, mmol/L	1.19 (0.88, 1.86)	1.26 (0.77, 1.71)	0.679
HDL, mmol/L	1.20 (1.04, 1.36)	1.12 (0.92, 1.36)	0.118
LDL, mmol/L	2.25 (1.75, 2.99)	2.29 (1.65, 2.88)	0.440
HbA1c, %	5.89 (5.46, 6.16)	5.80 (5.51, 6.28)	0.569
Hcy, umol/L	12.20 (10.06, 15.80)	14.40 (12.13, 16.38)	0.191
Total tau, pg/ml	169.4 (154.7, 181.6)	179.4 (169.0, 196.4)	<0.001*
P-tau181, pg/ml	43.72 (36.47, 59.75)	52.81 (38.35, 66.52)	0.017*
P-tau396, pg/ml	198.6 (139.4, 224.9)	188.9 (124.2, 292.7)	0.302
CRP, mg/L	0.82 (0.41, 1.42)	1.82 (0.73, 3.36)	<0.001*
IL-6, pg/mL	3.38 (2.79, 4.86)	4.53 (3.15, 6.55)	0.001*
IL-1β, pg/mL	4.34 (3.95, 5.04)	6.33 (5.10, 8.60)	<0.001*
IL-10, pg/mL	2.25 (1.29, 3.13)	2.12 (1.12, 3.29)	0.367
TNF-α, pg/mL	11.28 (8.82, 14.92)	13.26 (10.19, 18.04)	0.033*
IFN-γ, pg/mL	15.19 (10.32, 20.90)	18.42 (15.38, 26.87)	<0.001*

Data are presented as median (P25, P75), (*n* = 56 vs. *n* = 56), compared by covariance (ANCOVA), with age, gender, smoking status and stroke history as covariates. HCs, health controls; PSP, Progressive supranuclear palsy; TC, total cholesterol; TG, triglycerides; HDL, high-density lipoprotein cholesterol; LDL, low-density lipoprotein cholesterol; HbA1c, hemoglobin A1c; Hcy, homocysteine; CRP, C-reactive protein; IL-6, interleukin-6; IL-1β, interleukin-1β; IL-10, interleukin-10; TNF-α, tumor necrosis factor-α; IFN-γ, interferon γ.

*P < 0.05, presents a significant difference.

Progressive supranuclear palsy is a primary 4-repeat tauopathy characterized by abnormal accumulation of hyperphosphorylated tau in neurons and glia, particularly within the brainstem and basal ganglia. This pathology is strongly associated with MAPT mutations and the H1 haplotype, both of which promote tau misfolding and aggregation into neurofibrillary tangles and glial cytoplasmic inclusions (Forrest et al., 2023; Badihian et al., 2024; DeRosier et al., 2024). These pathological tau species are capable of propagating in a prion-like manner and are known to elicit neuroinflammatory responses, notably through microglial activation (Williams, 2006). Although current tau-related biomarkers—such as plasma and cerebrospinal fluid tau—lack disease specificity, they may hold diagnostic and mechanistic value in PSP (Giannakis et al., 2025). Based on this rationale, we assessed plasma levels of total tau, p-tau181, and p-tau396 in patients with PSP and HCs. After adjusting for age, gender, smoking status and stroke history, total tau (179.4 pg/mL vs. 169.4 pg/mL, *p* < 0.001) and p-tau181 (52.81 pg/mL vs. 43.72 pg/mL, *p* = 0.017) levels were elevated in patients with PSP compared to HCs, consistent with previous studies (Lin et al., 2018), whereas p-tau396 levels were not significantly different (Figures 1O–Q and Table 3). After adjustment for multiple covariates in multivariate regression models, higher plasma total tau levels showed positive association with carotid stenosis percentage (β = 0.240, *p* = 0.046). Similarly, elevated p-tau181 levels were independently associated with increased max-CPT (β = 0.434, *p* < 0.001), TPN (β = 0.392, *p* = 0.003), CPS (β = 0.460, *p* < 0.001), carotid stenosis% (β = 0.460, *p* < 0.001), and MRA-GSS (β = 0.293, *p* = 0.014) (Figure 1R and Supplementary Table 2). Collectively, these results indicate that increased plasma levels of both total tau and p-tau181 in patients with PSP are potentially associated with greater severity of atherosclerotic changes.

### 3.3 Increased proportion of macrophages in the peripheral blood of patients with PSP compared to HCs is accompanied by elevated plasma pro-atherosclerotic inflammatory cytokines

**FIGURE 2:**
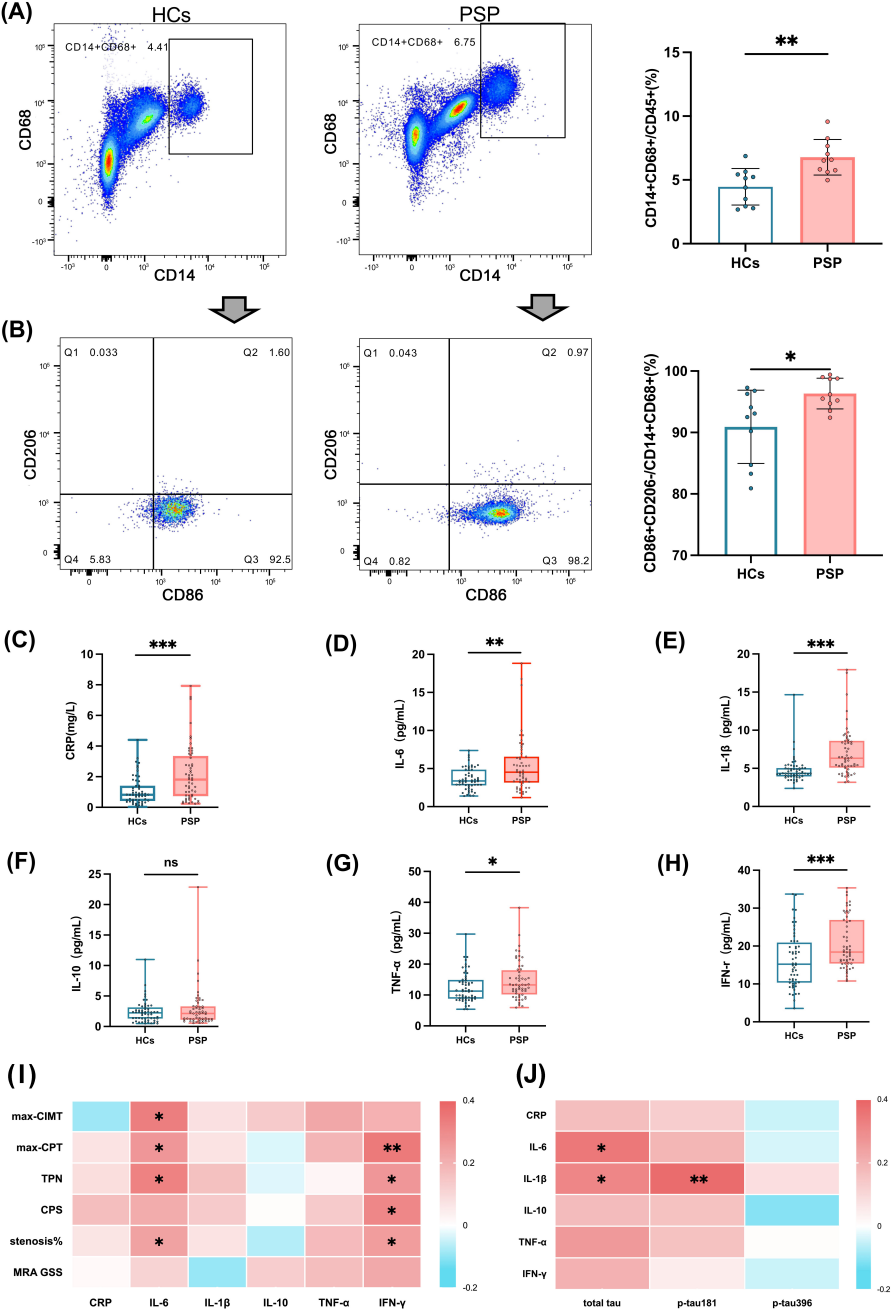
Elevated peripheral blood macrophage ratio and plasma inflammatory cytokine levels in patients with PSP. Representative flow cytometry analysis of **(A)** macrophages (CD14 + CD68+), and **(B)** classically activated macrophages (CD86 + CD206-M1) subpopulations from peripheral blood (left) and quantifications (right) in HCs (*n* = 10) and patients with PSP (*n* = 10). Plasma levels of inflammatory cytokine **(C)** CRP, **(D)** IL-6, **(E)** IL-1β, **(F)** IL-10, **(G)** TNF-α, and **(H)** IFN-γ in HCs (*n* = 56) and PSP with patients (*n* = 56). **(I)** Heatmap of multivariable linear regression heatmap showing the association between inflammatory cytokine levels (CRP, IL-6, IL-1β, IL-10, TNF-α, and IFN-r) and atherosclerosis measures (max-CIMT, max-CPT, TPN, CPS, carotid stenosis% and MRA GSS) in patients with PSP (*n* = 56). **(J)** Heatmap of multivariable linear regression heatmap showing the association between tau levels (total tau, p-tau181, p-tau396) and inflammatory cytokine levels (CRP, IL-6, IL-1β, IL-10, TNF-α, and IFN-r) in patients with PSP (*n* = 56). Results are expressed as mean and SD. Statistical approaches: **(A, B)** unpaired non-parametric Mann-Whitney U tests, **(C–H)** covariance (ANCOVA), with age, gender, smoking status and stroke history as covariates, **(I, J)** multivariable linear regression. In heatmaps, each cell represents the standardized regression coefficient (β) derived from multivariable linear regression models, where each *Y*-axis was regressed on *X*-axis while adjusting for age, gender, smoking history, hypertension, diabetes, and dyslipidemia. The color intensity of heatmap indicates the strength and direction of association (β), with positive associations shown in red and negative in blue. *P* < 0.05 indicates statistical significance (*p < 0.05, **p < 0.01, ***p < 0.001), and ns indicates *p* ≥ 0.05.

Emerging evidence indicates a potential link between tau pathology and vascular ischemia. In primary tauopathies, regions exhibiting tau accumulation often demonstrate abnormal microvascular architecture (Merlini et al., 2016). Tau proteins have been identified as key inducers of microglial activation and the subsequent release of pro-inflammatory mediators within the central nervous system (Gao et al., 2023). At the same time, macrophage infiltration and inflammation are well-established contributors to the pathogenesis of atherosclerosis (Blagov et al., 2023; Ajoolabady et al., 2024). Given these intersecting pathways, we investigated the percentage of peripheral blood macrophages and the plasma concentrations of inflammatory cytokines associated with atherosclerosis in both groups. Flow cytometric analysis of peripheral blood (*n* = 10 vs. *n* = 10) showed that the percentage of macrophages (CD14 + CD68+) in peripheral blood was significantly higher in patients with PSP compared to HCs (6.55% vs. 4.77%, *p* = 0.002), and the percentage of CD86 + CD206-M1 classically activated macrophages was increased as well (95.85% vs. 92.80%, *p* = 0.036) (Figures 2A, B). Analysis of the clinical data revealed that participants in the PSP group had higher plasma CRP levels (1.59 mg/L vs. 0.84 mg/L, *p* < 0.001) (*n* = 56 vs. *n* = 56) (Figure 2C and Table 3), which suggests a systemic inflammatory response in patients with PSP. In the assay of plasma inflammatory cytokines, IL-6 (4.53 pg/mL vs. 3.38 pg/mL, *p* = 0.001), IL-1β (6.33 pg/mL vs. 4.34 pg/mL, *p* < 0.001), TNF-α(13.26 pg/mL vs. 11.28 pg/mL, *p* = 0.033) and IFN-γ (18.42 pg/mL vs. 15.19 pg/mL, *p* < 0.001) were elevated in patients with PSP compared to HCs (*n* = 56 vs. *n* = 56), whereas there was no significant difference in the levels of anti-atherosclerotic cytokine IL-10 (2.12 pg/mL vs. 2.25 pg/mL, *p* = 0.367) after adjusting for age, gender, smoking status and stroke history (Figures 2D–H and Table 3).

Subsequent multivariate linear regression models revealed that elevated IL-6 levels were independently associated with increased max-CIMT (β = 0.331, *p* = 0.013), max-CPT (β = 0.265, *p* = 0.044), TPN (β = 0.322, *p* = 0.014), and carotid stenosis% (β = 0.243, *p* = 0.044). Similarly, higher IFN-γ levels correlated with greater max-CPT (β = 0.341, *p* = 0.007), TPN (β = 0.265, *p* = 0.040), CPS (β = 0.307, *p* = 0.010), and carotid stenosis percentage (β = 0.251, *p* = 0.032) (Figure 2I and Supplementary Table 3). Further multivariate linear regression revealed positive correlations of total tau with IL-6 (β = 0.346, *p* = 0.014) and IL-1β (β = 0.312, *p* = 0.028), and of p-tau181 with IL-1β (β = 0.379, *p* = 0.008) in patients with PSP (Figure 2J and Supplementary Table 4). These results demonstrate that elevated plasma IL-6 levels in PSP patients show significant positive correlations with both multiple atherosclerosis indices and total tau levels, suggesting a potential mechanistic link between tau-associated systemic inflammation and accelerated atherosclerosis in this population.

### 3.4 Aortic wall thickening in tau-PFFs-injected mice is accompanied by increased macrophages and elevated inflammatory cytokines

**FIGURE 3:**
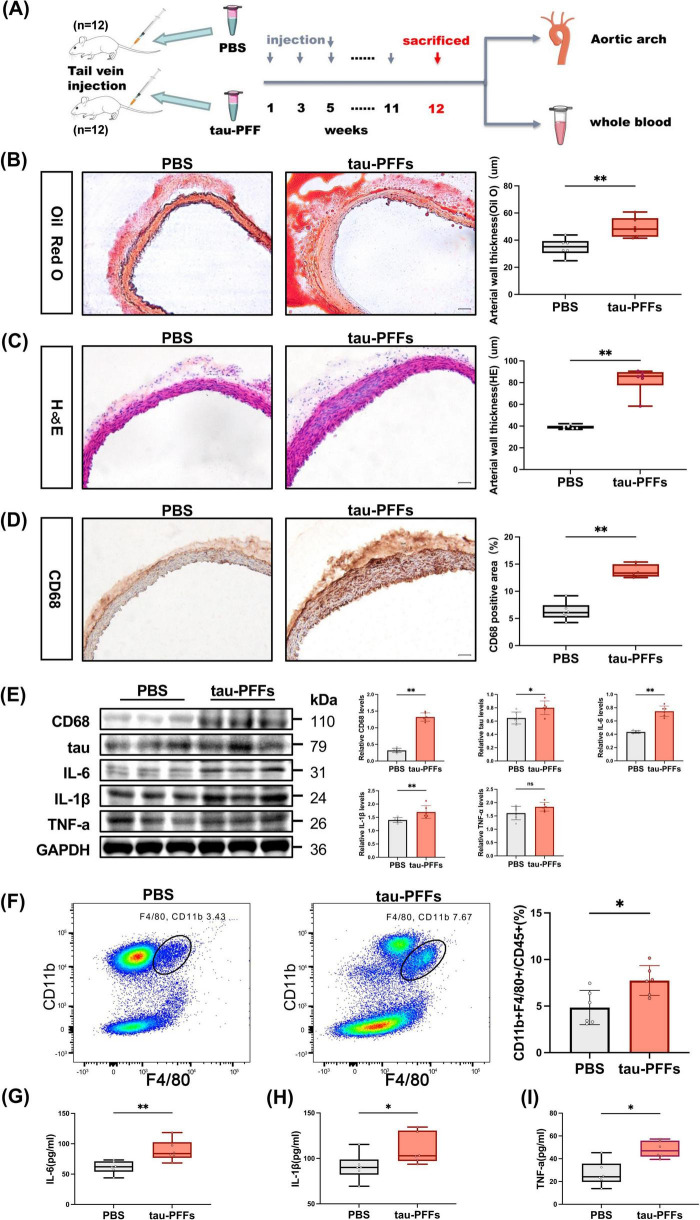
Mouse model confirms impact of tau-PFFs on atherosclerosis development. **(A)** The flow chart of the animal experiment. Representative image of **(B)** Oil Red O staining and **(C)** H&E staining of the aortic arch to detect morphologic changes in the vessel wall (left) and quantifications (right). Representative images of immunohistochemical detection of **(D)** macrophages (CD68) in the aorta arch (left) and quantifications (right). Scale bar: 50 μm. **(E)** Representative immunoblot images of CD68, tau, IL-6, IL-1β, and TNF-a in the aorta (left) and quantifications (right). The loading controls (GAPDH) were run on different gels in the same experiment (*n* = 6). **(F)** Representative flow cytometry analysis of macrophages (CD11b + F4/80+) subpopulations from peripheral blood (left) and quantifications (right) of PBS (*n* = 6) and tau-PFFs groups (*n* = 6). **(G–I)** Plasma levels of inflammatory cytokine (IL-6, IL-1β, and TNF-α) in PBS (*n* = 6) and tau-PFFs groups (*n* = 6). Results are expressed as mean and SD. Statistical approaches: **(B–I)** unpaired non-parametric Mann-Whitney U tests. *P* < 0.05 indicates statistical significance (*p < 0.05, **p < 0.01), and ns indicates *p* ≥ 0.05.

To investigate the role of tau in the activation of peripheral blood macrophages to release inflammatory factors and atherosclerotic processes, we injected tau-PFFs into the tail vein to increase the peripheral blood tau protein concentration in C57BL/6J mice, while the control group was injected with sterile PBS. Aortic tissue and blood were collected 3 months after the start of the injection (Figure 3A). Oil Red O and H&E staining of the aortic arch in the tau-PFF group revealed heterogeneous thickening of the arterial wall with structural disorganization and increased cell numbers without atherosclerotic plaque formation (Figures 3B, C). Since CD68 is a marker of macrophages, the increased CD68 expression, as assessed by immunohistochemical staining and western blotting (*p* = 0.002), suggested macrophage infiltration into the arterial wall in the tau-PFF groups (Figures 3D, E). In addition, western blot analysis revealed significantly upregulated expression of tau (*p* = 0.015), IL-6 (*p* = 0.002), and IL-1β (*p* = 0.004) in the tau-PFFs groups compared to controls (Figure 3E), confirming enhanced tau deposition and inflammatory responses within the arterial wall. Similarly, flow cytometric analysis revealed enhanced macrophage subpopulations (CD11b + F4/80+) (7.75% vs. 4.41%, *p* = 0.026) in the peripheral blood of the tau-PFFs group compared to the control group (*n* = 6 vs. *n* = 6) (Figure 3F). ELISA of murine plasma inflammatory cytokines revealed elevated levels of IL-6 (*p* = 0.009), IL-1β (*p* = 0.026), and TNF-α (*p* = 0.015) in the tau-PFFs group compared to the control group, indicating an enhanced tau-induced inflammatory response in the peripheral blood (Figure 3G–I).

## 4 Discussion

In the present study, we identified a greater degree of atherosclerosis in patients with PSP than in HCs. In addition, patients with PSP had increased macrophage proportions and elevated levels of pro-atherosclerotic inflammatory factors, which were positively correlated with total plasma tau levels and the degree of atherosclerosis. Animal studies demonstrated that chronic tau-PFFs administration via tail vein injection significantly enhanced aortic macrophage infiltration and inflammatory responses, accompanied by localized intimal thickening - a potential precursor of atherosclerotic lesions. Our study contributes to the understanding of the association between PSP and atherosclerotic disease and provides evidence for the etiology of high cerebrovascular morbidity in patients with PSP.

Carotid atherosclerosis is a reflection of systemic atherosclerosis associated with systemic vascular disease and is strongly associated with new-onset ischemic stroke (Faxon et al., 2004). MRA is a validated technique for the assessment of intracranial vascular stenosis with a sensitivity for the correct diagnosis of intracranial stenosis and a specificity of 70% and 99%, respectively (Bash et al., 2005). Our study confirmed the presence of a heavier carotid and intracranial atherosclerotic burden in patients with PSP based on the results of carotid ultrasonography and MRA, indicating a higher susceptibility to cerebrovascular disease.

The measurement of plasma tau showed that the total tau and p-tau181 levels in patients were higher than those in HCs, which was positively correlated with the degree of atherosclerosis. A prior study utilizing the Simoa kit to measure plasma total-tau levels in 8 PSP patients and 33 healthy controls reported no significant intergroup differences (Li et al., 2022). However, disparities in sample sizes and baseline clinical characteristics between two studys may have limited statistical power. These findings highlight the need for large-scale, multicenter studies with matched cohorts in order to definitively assess changes in plasma tau in PSP. The role of tau proteins in vascular disease is not fully understood, but a previous study found a positive correlation between the severity of cerebral amyloid angiopathy and tau burden (Rabin et al., 2022). In this study, we hypothesized that tau plays a driving role in atherosclerosis.

An increased percentage of classical monocytes in peripheral blood and significant differences in serum IL-1β and IL-6 levels in patients with PSP compared to HCs have been reported in a few studies (Grozdanov et al., 2014; Madetko-Alster et al., 2023). It is well known that peripheral blood macrophages not only secrete inflammatory factors but also differentiate into foam cells in the aortic endothelium, which play an important role in the atherosclerotic process (Tabas and Bornfeldt, 2016). In our study, we observed elevated levels of macrophages and pro-atherosclerotic inflammatory factors in the peripheral blood of patients with PSP, which may be associated with tau. Tau proteins are major inducers of microglial activation in the brains of patients with PSP (Gao et al., 2023). Microglia, which act as macrophages of the central nervous system, phagocytose various tau proteins, become activated, and release inflammatory factors (Majerova et al., 2014; Bolós et al., 2016). Immunochemical analysis of brain tissue from patients with PSP showed activation and increased numbers of microglia in the substantia nigra compacta, subthalamic nucleus, pyramidal, and extrapyramidal motor systems, which correlated positively with tau burden, and upregulated expression levels of pro-inflammatory factors such as IL-1β were found in the substantia nigra (Gao et al., 2023). Similarly, our findings support a potential role of inflammation in PSP pathogenesis. Current evidence suggests PSP arises from multifactorial interactions between genetic susceptibility (notably MAPT variants affecting tau metabolism) and environmental influences (e.g., chronic inflammation) (Müller et al., 2025). Inflammation is both a consequence of tau pathology and may accelerate disease progression through positive feedback loops (Darricau et al., 2023; Madetko-Alster et al., 2023; Muñoz-Delgado et al., 2024). Future studies are needed to further clarify the molecular associations between specific genetic variants and inflammatory pathways. Moreover, enhanced peripheral inflammatory responses have been observed in various neurodegenerative diseases, including PD and AD (Muñoz-Delgado et al., 2023; Walker et al., 2023). Peripheral inflammation exacerbates the progression of these diseases through multiple pathways, including immune cell infiltration, cytokine release and metabolite effects, and it exhibits heterogeneity in different diseases (Dias-Carvalho et al., 2024; Asimakidou et al., 2025; Oyamada et al., 2025). While interventions targeting peripheral inflammation may offer new avenues for treatment, a deeper understanding of the underlying mechanisms and their clinical translation is still required.

A previous study demonstrated that the addition of tau oligomers and fibrils to primary microglial cell cultures induced microglial activation and the secretion of pro-inflammatory cytokines (Morales et al., 2013). Our animal data suggest that increased pathological tau in the peripheral blood induces macrophage proliferation, enhances inflammatory responses in peripheral tissues, and may also induce the development of atherosclerosis. However, we did not find typical atherosclerotic plaques, which may require a longer period of time or a more intense stimulus. We found increased tau expression in the aorta. Previous studies have shown that tau oligomers accumulate in the cerebral microvasculature of patients with AD and PSP (Castillo-Carranza et al., 2017). Although the specific role of tau accumulation in atherosclerosis is unknown, it may represent a novel mechanism by which the functional and structural integrity of blood vessels is compromised. This potential tau-atherosclerosis interaction aligns with established mechanisms whereby CNS-derived tau proteins may amplify peripheral inflammation through microglial activation and blood-brain barrier dysfunction (Hossen et al., 2024; Kim et al., 2024), though causal relationships require further longitudinal validation.

However, this study had a few limitations. First, in terms of clinical studies, the participants were from a single center; future multicenter studies are needed to confirm these results. In addition, this study included patients with a clinical diagnosis of probable or possible PSP but no pathological diagnosis. Given that neuropathological examination remains the diagnostic gold standard for PSP, future examination of brain, vascular, and systemic tissues from postmortem PSP cases could help confirm the presence and distribution of tau pathology, as well as associated vascular inflammation and structural changes, such as endothelial activation, intimal thickening, or immune cell infiltration. Integrating neuropathological data with ante-mortem imaging and biomarker profiles may further enhance our understanding of the tau–inflammation and its role in PSP. Secondly, while our current study focused on systemic and vascular responses to repeated tau-PFFs exposure, further work is needed to clarify whether these changes translate into functional motor impairments relevant to PSP. Additionally, histological analysis of central motor regions will help determine whether vascular damage co-localizes with tau pathology. These efforts will provide a more integrated understanding of the tau in disease progression and improve the mechanistic relevance of this model.

In summary, this study indicated that patients with PSP are more susceptible to cerebrovascular disease and have an enhanced inflammatory response in the circulatory system. Pathological tau contributes to macrophage proliferation and inflammation, potentially promoting atherosclerosis development. Clinicians should consider screening patients with PSP for atherosclerosis and monitoring inflammatory markers to mitigate cardiovascular risk. Targeting tau pathology and associated inflammation may offer a promising therapeutic strategy for reducing cardiovascular risk in patients with PSP. Overall, our findings underscore the importance of recognizing and addressing vascular complications in PSP to enhance patient outcomes.

## Data Availability

The original contributions presented in this study are included in this article, raw data files are available from the corresponding author upon reasonable request.
