# Supplementary material for: Increased tau-induced inflammatory responses are associated with a greater degree of atherosclerosis in progressive supranuclear palsy
Source: Front Aging Neurosci. 2025 Aug 11;17:1608631. doi: 10.3389/fnagi.2025.1608631 (PMC12375634; doi:10.3389/fnagi.2025.1608631)
Supplement: Supplementary file 1 [file Table_1.docx]

**Supplementary Table 1 Multivariable linear regression results for associations between clinical parameters and vascular atherosclerosis measures in patient with PSP**

|  | Duration | H&Y staging | UPDRS-3 | PSPRS | MMSE |
| --- | --- | --- | --- | --- | --- |
| max-CIMT | β=0.099  *P*=0.439 | β=0.328  *P*=0.015* | β=0.371  *P*=0.007* | β=0.251  *P*=0.073 | β=0.034  *P*=0.844 |
| max-CPT | β=0.152  *P*=0.222 | β=0.331  *P*=0.012* | β=0.288  *P*=0.033* | β=0.256  *P*=0.061 | β=-0.053  *P*=0.750 |
| TPN | β=0.348  *P*=0.004* | β=0.272  *P*=0.043* | β=0.193  *P*=0.166 | β=0.158  *P*=0.259 | β=-0.084  *P*=0.621 |
| CPS | β=0.203  *P*=0.082 | β=0.300  *P*=0.016* | β=0.278  *P*=0.030* | β=0.270  *P*=0.036* | β=-0.101  *P*=0.516 |
| stenosis% | β=0.192  *P*=0.091 | β=0.256  *P*=0.036* | β=0.211  *P*=0.092 | β=0.147  *P*=0.247 | β=-0.099  *P*=0.517 |
| MRA GSS | β=-0.044  *P*=0.700 | β=0.078  *P*=0.527 | β=0.082  *P*=0.513 | β=-0.008  *P*=0.951 | β=0.089  *P*=0.561 |

Note: Standardized regression coefficients (β) and corresponding *p*-values are shown for each regression model. Each atherosclerosis outcome (max-CIMT, max-CPT, TPN, CPS, carotid stenosis% and MRA GSS) was used as the dependent variable in a separate model, and each clinical parameter (disease duration, H&Y staging scale, UPDRS-Ⅲ, PSPRS, MMSE) was included as an independent variable. All models were adjusted for age, sex, smoking history, hypertension, diabetes, dyslipidemia, and stroke history (for MMSE, one more correction for period of education). **p* < 0.05, presents a significant difference.

**Supplementary Table 2 Multivariable linear regression results for associations between tau levels and vascular atherosclerosis measures in patient with PSP**

|  | total tau | p-tau181 | p-tau396 |
| --- | --- | --- | --- |
| max-CIMT | β=0.251  *P*=0.061 | β=0.225  *P*=0.100 | β=0.110  *P*=0.446 |
| max-CPT | β=0.230  *P*=0.080 | β=0.434  *P*<0.001* | β=0.216  *P*=0.122 |
| TPN | β=0.220  *P*=0.099 | β=0.392  *P*=0.003* | β=0.216  *P*=0.125 |
| CPS | β=0.223  *P*=0.073 | β=0.436  *P*<0.001* | β=0.194  *P*=0.142 |
| stenosis% | β=0.240  *P*=0.046* | β=0.460  *P*<0.001* | β=0.141  *P*=0.272 |
| MRA GSS | β=0.141  *P*=0.243 | β=0.293  *P*=0.014* | β=0.088  *P*=0.493 |

Note: Standardized regression coefficients (β) and corresponding *p*-values are shown for each regression model. Each atherosclerosis outcome (max-CIMT, max-CPT, TPN, CPS, carotid stenosis% and MRA GSS) was used as the dependent variable in a separate model, and each tau levels (total tau, p-tau181, p-tau396) was included as an independent variable. All models were adjusted for age, sex, smoking history, hypertension, diabetes, dyslipidemia, and stroke history.* *p* < 0.05, presents a significant difference.

**Supplementary Table 3 Multivariable linear regression results for associations between inflammatory cytokine levels and vascular atherosclerosis measures in patient with PSP**

|  | CRP | IL-6 | IL-1β | IL-10 | TNF-α | IFN-γ |
| --- | --- | --- | --- | --- | --- | --- |
| max-CIMT | β=-0.088  *P*=0.567 | β=0.331  *P*=0.013* | β=0.076  *P*=0.581 | β=0.136  *P*=0.342 | β=0.227  *P*=0.086 | β=0.209  *P*=0.114 |
| max-CPT | β=0.072  *P*=0.634 | β=0.265  *P*=0.044* | β=0.073  *P*=0.587 | β=-0.033  *P*=0.812 | β=0.187  *P*=0.150 | β=0.341  *P*=0.007* |
| TPN | β=0.084  *P*=0.582 | β=0.322  *P*=0.014* | β=0.160  *P*=0.236 | β=-0.031  *P*=0.828 | β=0.022  *P*=0.866 | β=0.265  *P*=0.040* |
| CPS | β=0.167  *P*=0.239 | β=0.212  *P*=0.090 | β=0.136  *P*=0.283 | β=0.004  *P*=0.976 | β=0.137  *P*=0.265 | β=0.307  *P*=0.010* |
| stenosis% | β=0.068  *P*=0.623 | β=0.243  *P*=0.044* | β=0.073  *P*=0.555 | β=-0.067  *P*=0.603 | β=0.176  *P*=0.139 | β=0.251  *P*=0.032* |
| MRA GSS | β=0.015  *P*=0.914 | β=0.113  *P*=0.352 | β=-0.092  *P*=0.453 | β=0.130  *P*=0.308 | β=0.165  *P*=0.161 | β=0.220  *P*=0.059 |

Note: Standardized regression coefficients (β) and corresponding p-values are shown for each regression model. Each atherosclerosis outcome (max-CIMT, max-CPT, TPN, CPS, carotid stenosis% and MRA GSS) was used as the dependent variable in a separate model, and each inflammatory cytokine levels (CRP, IL-6, IL-1β, IL-10, TNF-α, and IFN-r) was included as an independent variable. All models were adjusted for age, sex, smoking history, hypertension, diabetes, dyslipidemia, and stroke history.* *p* < 0.05, presents a significant difference.

**Supplementary Table 4 Multivariable linear regression results for associations between tau levels and inflammatory cytokine levels in patient with PSP**

|  | total tau | p-tau181 | p-tau396 |
| --- | --- | --- | --- |
| CRP | β=0.167  *P*=0.191 | β=0.129  *P*=0.323 | β=-0.047  *P*=0.732 |
| IL-6 | β=0.346  *P*=0.014* | β=0.190  *P*=0.195 | β=-0.039  *P*=0.802 |
| IL-1β | β=0.312  *P*=0.028* | β=0.379  *P*=0.008* | β=0.082  *P*=0.595 |
| IL-10 | β=0.174  *P*=0.209 | β=0.159  *P*=0.257 | β=-0.109  *P*=0.457 |
| TNF-α | β=0.260  *P*=0.077 | β=0.157  *P*=0.295 | β=0.003  *P*=0.984 |
| IFN-γ | β=0.201  *P*=0.175 | β=0.048  *P*=0.753 | β=-0.048  *P*=0.761 |

Note: Standardized regression coefficients (β) and corresponding p-values are shown for each regression model. Each inflammatory cytokine levels (CRP, IL-6, IL-1β, IL-10, TNF-α, and IFN-r) was used as the dependent variable in a separate model, and each tau levels (total tau, p-tau181, p-tau396) was included as an independent variable. All models were adjusted for age, sex, smoking history, hypertension, diabetes, dyslipidemia, and stroke history.* *p* < 0.05, presents a significant difference.
